# Selection of Stable Reference Genes for QRT-PCR in Tree Peony ‘Doulv’ and Functional Analysis of *PsCUC3*

**DOI:** 10.3390/plants13131741

**Published:** 2024-06-24

**Authors:** Shuang Zhou, Chao Ma, Wenbin Zhou, Shuangcheng Gao, Dianyun Hou, Lili Guo, Guoan Shi

**Affiliations:** College of Agriculture, Henan University of Science and Technology, Luoyang 471023, China; zhoushuang2001@163.com (S.Z.); machao840508@163.com (C.M.); zwl26088@163.com (W.Z.); gsczml@163.com (S.G.); dianyun518@163.com (D.H.); guolili0928@126.com (L.G.)

**Keywords:** ‘Doulv’ peony, reference gene, *PsCUC3*, functional analysis, floral type mutation

## Abstract

(1) Background: Tree peonies display extensive cultivar diversity due to widespread hybridization, resulting in a complex genetic architecture. This complexity complicates the selection of universal reference genes across different cultivars for qRT-PCR analyses. *Paeonia suffruticosa* ‘Doulv’, notable for its unique green blooms in China, exhibits chlorosis post-flowering and features petaloid stamens and pistils. (2) Methods: Based on published literature and RNA-seq data from ‘Doulv’, nine candidate reference genes—*ACT* (*Actin*), *TUB* (*β-Tubulin*), *UBC* (*Ubiquitin Conjugating Enzyme*), *UBQ* (*Ubiquitin*), *UPL* (*Ubiquitin Protein Ligase*), *PP2A* (*Protein Phosphatase 2A*), *PP2C* (*Protein Phosphatase 2C*), *MBF1A* (*Multiprotein Bridging Factor 1A*), and *GAPDH* (*Glyceraldehyde-3-Phosphate Dehydrogenase*)—were selected. Their expression stability was assessed across various tissues and developmental stages of ‘Doulv’ flowers using qRT-PCR, with evaluations conducted via GeNorm_v3.5, NormFinder_v20, and BestKeeper_v1.0. Gene cloning and expression analyses of *PsCUC3*, including its subcellular localization, were performed. (3) Results: *GAPDH* and *ACT* were identified as the most stable reference genes in petaloid stamens across various developmental stages of ‘Doulv’, whereas *UBC* and *MBF1A* were optimal across different tissues. Notably, specific conserved amino acids in PsCUC3 from ‘Doulv’ diverged from those in NAM/CUC3 proteins of other species, impacting its protein structure. *PsCUC3* expression analysis revealed no correlation with chlorophyll content in petaloid stamens but an association with petaloid organ development. Furthermore, PsCUC3 was predominantly localized in the nucleus. (4) Conclusions: This study comprehensively evaluated suitable reference genes using GeNorm_v3.5, NormFinder_v20, and BestKeeper_v1.0 software, establishing a robust qRT-PCR detection system for ‘Doulv’ peony. These results provide a solid experimental foundation for further research on ‘Doulv’ peony. Building on this experimental foundation, the functional analysis of the *PsCUC3* gene was conducted. The findings suggest a potential association between the *PsCUC3* gene and floral morphology alterations in ‘Doulv’, identifying *PsCUC3* as crucial for understanding the molecular mechanisms influencing floral structure in tree peonies.

## 1. Introduction

Tree peonies, scientifically known as *Paeonia suffruticosa* Andrews, are indigenous to China and boast a heritage extending over 1400 years. Renowned for their large, exquisite blooms, these peonies are celebrated as the ‘king of flowers’, symbolizing prosperity and joy in Chinese culture. China is home to over 1000 cultivars of tree peonies [1]. The flower colors of tree peonies can be divided into nine types: red, pink, yellow, purple, green, white, black, blue, and multi-color. Most research on tree peonies has focused on red, pink, yellow, purple, and multi-color blooms [2,3,4,5,6,7,8,9] and petal blotch [10,11], which are bright and conspicuous, with no prior studies reported on green-colored peonies. There are more than ten cultivars of green flower peonies in China, but all their blooms transition to white or pink upon opening, significantly diminishing their ornamental appeal. The molecular mechanisms underlying flower chlorosis in green flower peonies remain unclear.

Globally, there are more than 2000 cultivars of tree peonies, each resulting from extensive hybridization during cultivar development, leading to a complex genetic makeup. Lv et al. mapped four publicly available RNA-seq datasets to the draft genome of the tree peony cultivar “Luo shen xiao chun”, and the mapping ratios were very low, ranging from 0.04% to 68.41% [12], indicating high genetic diversity among tree peony cultivars and the lack of universality among reference genes for qRT-PCR analysis. Zhou et al. analyzed the expression of 12 candidate reference genes across various tissues and five developmental stages of tree peony in six distinct colors using qPCR. Their findings identified *PP2A*, *UPL*, and *UBQ* as the most suitable reference genes across all tested samples. *Helicase*, *TUA* (*alpha-tubulin*), and *EIF5A* (*eukaryotic translation initiation factor 5A*) also exhibited notable expression stability across different samples [13]. In the cultivars ‘Feng Dan’ and ‘Xi Shi’, *GAPDH* and *UBC* were highlighted as optimal reference genes, whereas *EF-1α* (*elongation factor 1α*) and *UBC* showed the greatest stability in ‘Que Hao’ [14]. In *Paeonia ostii*, *UBQ* and *Tip41* (*tip41-like protein*) were the most stable reference genes across various tissues and seed developmental stages [15]. The genes *ERVTP* (*endoplasmic reticulum vesicle transporter protein*) and *PP2CFP* (*protein phosphatase 2C family protein*) were identified as the best for petals during different stages of floral development in *Paeonia ostii* cultivars ‘Feng Dan’, ‘Xi Shi’, and ‘Que Hao’. *RPS9* (*ribosomal protein S9*) and *ARFA1C* (*ADP-ribosylation factor A1C*) maintained high expression stability across flowers of different colors in ‘Feng Dan’. Similarly, *AMPDS* (*AMP-dependent synthetase family protein*) and *PUF1639* (*protein of unknown function 1639*) showed stable expression across various tissues, with *RPS9* and *PUF1639* being the most stable in seeds at different developmental stages. *PUF1639*, *MBF1A*, *PP2CFP*, and *RPS9* outperformed traditional reference genes in tree peony [16]. In the Itoh peony ‘Bartzella’, *UBQ* and *TIP41* were the best reference genes for petals at different developmental stages, whereas *β-TUB*, *UBQ*, and *ACT* were the most stable in various organs [17]. It is necessary to screen stable reference genes when conducting gene expression analysis in previously unreported peony varieties.

*Paeonia suffruticosa* ‘Doulv’ is the most famous tree peony cultivar in China, particularly notable for its unique green flowers, which are considered precious and rare in China. Initially green during the early flowering stages, the blooms of ‘Doulv’ transition to white upon opening. The presence of petaloid stamens and pistils gives the ‘Doulv’ peony a crown-like or globular floral morphology, making it an excellent candidate for exploring the molecular mechanisms involved in flower type development in tree peonies. The selection of appropriate reference genes was a necessary experimental foundation for further study of the molecular mechanisms underlying flower chlorosis and flower type formation in ‘Doulv’ peony. In this study, stable reference genes were comprehensively evaluated using GeNorm_v3.5, NormFinder_v20, and BestKeeper_v1.0 software, and a robust qRT-PCR detection system for ‘Doulv’ peony was successfully established. Based on this experimental foundation, functional analysis of the *PsCUC3* gene was carried out. *CUP-SHAPED COTYLEDONs* (*CUCs*) are members of the NAC transcription factor family, pivotal in pattern formation [18], organ separation [19], organ boundary formation [20], and floral development [21,22]. Comprehensive methodologies including gene cloning, expression analysis, and subcellular localization of *PsCUC3* were executed. These procedures have established a foundational platform for subsequent inquiries into the molecular mechanisms influencing floral morphology in ‘Doulv’ peony.

## 2. Results

### 2.1. Flower Characteristics of ‘Doulv’ Peony

**Figure 1:**
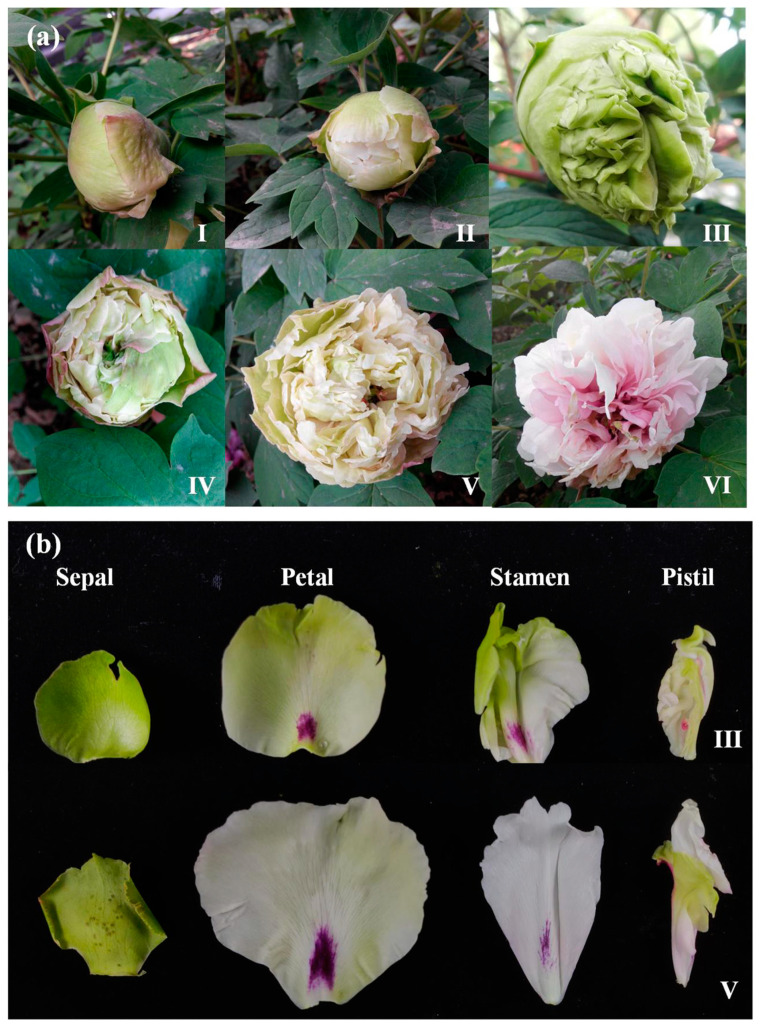
Floral characteristics of ‘Doulv’ peony. (**a**) Developmental stages of ‘Doulv’ flowers. Stage I: sepals crack open, exposing the outer petals. Stage II: inner petals become visible as the buds fluff up. Stage III: outer petals fully expand, inner petals loosen, though the innermost petals remain closed. Stage IV: inner petals expand fully, revealing the carpels. Stage V: all inner petals are fully expanded, with carpels completely exposed. Stage VI: outer petals start to dehydrate and undergo senescence. (**b**) Tissue differentiation in ‘Doulv’ flower at Stage III and Stage V. Stage III features fully expanded outer petals and loosened inner petals, while innermost petals are still closed, maintaining overall green coloration. By Stage V, all inner petals are fully expanded, and carpels are entirely exposed, with a shift from green to white in color.

The floral morphology of ‘Doulv’ peony is characterized by a crown-like or globular shape. Externally, the flowers consist of intact petals, while internally, they feature petaloid stamens and pistils. During Stage III, the blooms of ‘Doulv’ exhibit a distinct green hue, transitioning to white by Stage V (Figure 1a). Throughout the blooming period, the sepals, petals, and petaloid pistils maintain their green coloration. In contrast, the petaloid stamens, which constitute a significant portion of the ‘Doulv’ floral structure, exhibit chlorosis following the flower’s opening (Figure 1b).

### 2.2. Gene-Specific PCR Amplification Efficiency Analysis

**Figure 2:**
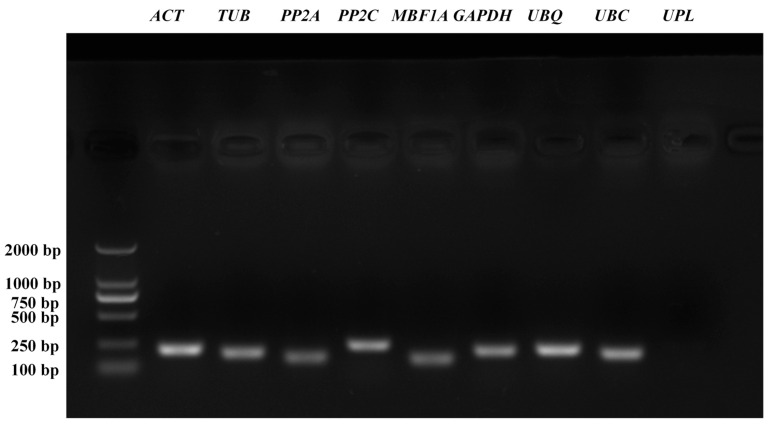
The agarose gel electrophoresis map of candidate reference genes in ‘Doulv’.

**Figure 3:**
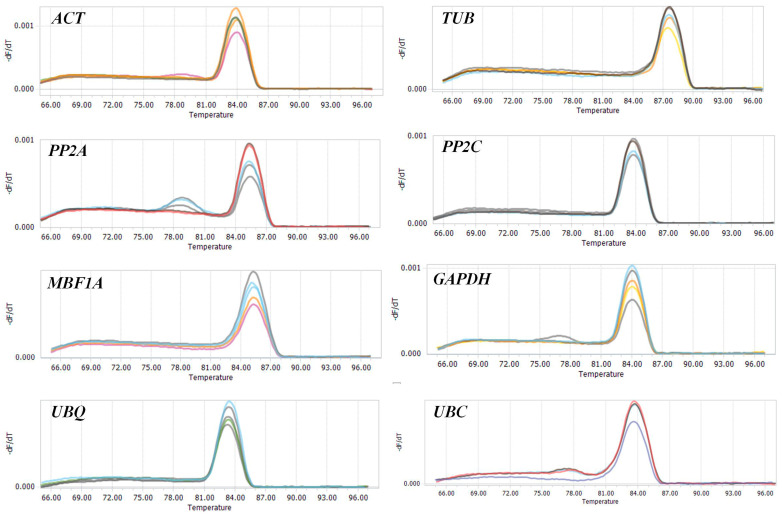
Melting curve of candidate reference genes.

RT-PCR amplification results revealed specificity in nine candidate reference genes, with the exception of *UPL*, which displayed no band formation. The specific PCR products for the remaining eight genes presented as single bands at expected lengths (Figure 2), qualifying them for further analysis. Additionally, the melting curves for each primer set exhibited a single peak, as shown in Figure 3. The amplification efficiency and correlation coefficients (*R*^2^) were determined from the standard curve slope. The *R*^2^ values for all detected candidate reference genes ranged from 0.9911 to 0.9979. However, the amplification efficiency of *PP2A* was 165.38%, exceeding the optimal threshold of 105%, rendering it unsuitable for qRT-PCR. The amplification efficiencies of the other seven genes were between 90.63% and 102.94%, with amplicon sizes ranging from 140 to 233 bp. These seven genes were deemed appropriate for qRT-PCR analyses and were utilized to assess the stability of reference genes (Table 1).

### 2.3. Expression Stability Analysis of Candidate Reference Genes

**Figure 4:**
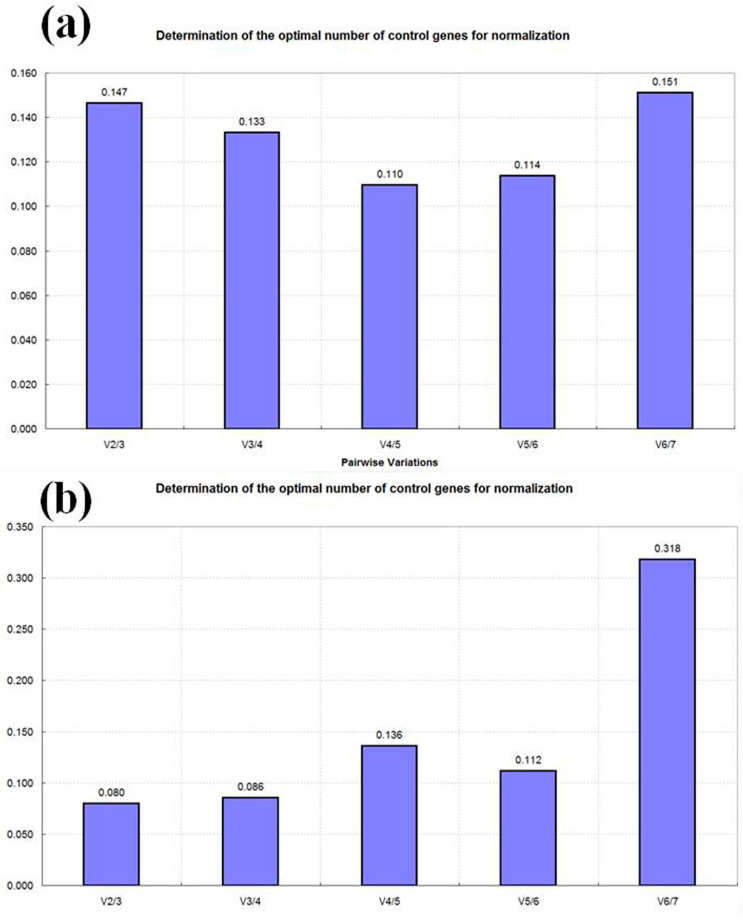
Paired difference analysis (V*n*/*n* + 1) of candidate reference genes by GeNorm. (**a**) Pairwise variation value (V value) of reference genes in petaloid stamens at different developmental stages of ‘Doulv’; (**b**) V value of reference genes in different tissues of ‘Doulv’ flower.

The expression stability of candidate reference genes was analyzed using GeNorm_v3.5, NormFinder_v20, and BestKeeper_v1.0 software, as per Zhang et al. [23]. A geomean of ranking values, derived from M values (GeNorm), S values (NormFinder), and SD (BestKeeper), was utilized to comprehensively assess the expression stability of these genes. Additionally, paired difference analysis (V*n*/*n + 1*) conducted by GeNorm determined the optimal number of internal control genes. As demonstrated in Figure 4a, for the petaloid stamens at different developmental stages of ‘Doulv’, a V2/3 value less than 0.15 indicated that two reference genes were sufficient for qRT-PCR. *GAPDH* and *ACT* were identified as the most stable reference genes for these stages (Table 2). Similarly, in different tissues of the ‘Doulv’ flower, a V2/3 value less than 0.15 (Figure 4b) suggested the necessity of two reference genes. *UBC* and *MBF1A* were chosen as the optimal internal reference gene combination for these tissues (Table 3).

### 2.4. Cloning and Bioinformatics Analysis of PsCUC3 from ‘Doulv’ Peony

**Figure 5:**
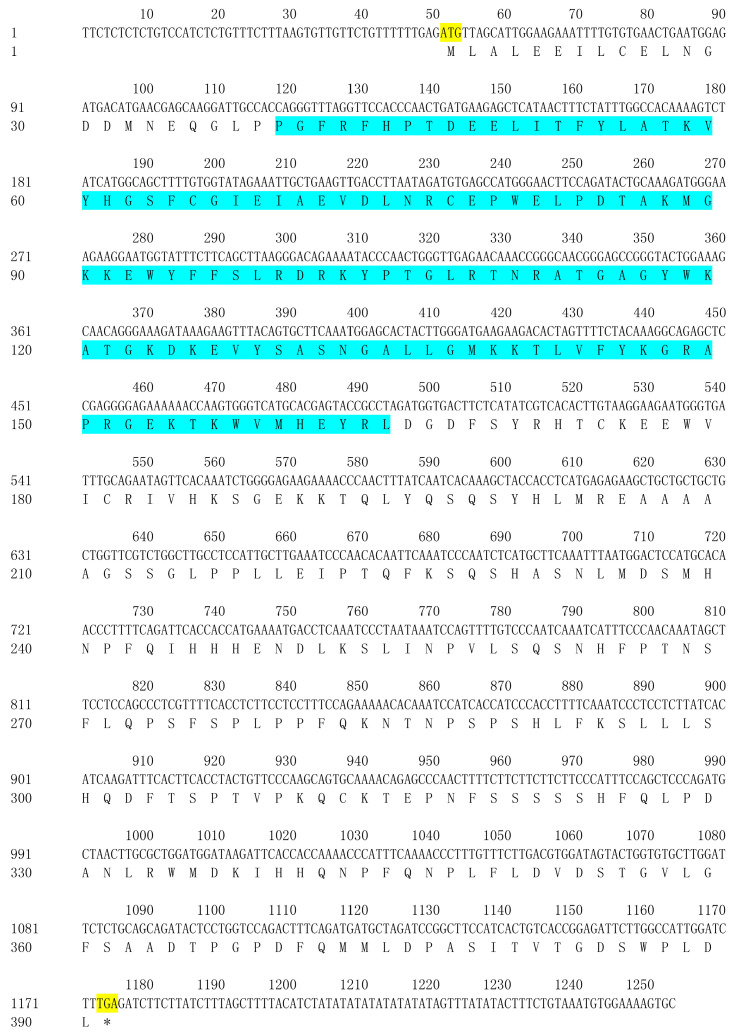
Nucleotide sequence and deduced amino acid sequence of the *PsCUC3* from ‘Doulv’ peony. Yellow: initiation codon and termination codon, respectively. Blue: NAC domain (23-148 aa). *: biosynthesis termination of PsCUC3 protein.

The full-length cDNA of *PsCUC3* spans 1253 bp (Figure S1), encompassing an open reading frame (ORF) of 1125 bp that encodes an NAC protein consisting of 374 amino acids (Figure 5). The *PsCUC3* gene has been deposited in GenBank under the accession number OP745631.

The molecular weight of the PsCUC3 protein is calculated at 42.38 kDa, with a theoretical isoelectric point (pI) of 6.38. The stability of the PsCUC3 protein is suggested by an instability index of 39.83, classifying it as stable. The protein’s aliphatic index stands at 65.75, indicating robust thermostability, and the grand average of hydropathicity (GRAVY) at −0.560 confirms its hydrophilic nature. The secondary structure of PsCUC3 comprises 24.33% alpha helices, 16.04% extended strands, 5.61% beta turns, and 54.01% random coils. Predictive analyses suggest that the PsCUC3 protein localizes primarily within the nucleus.

**Figure 6:**
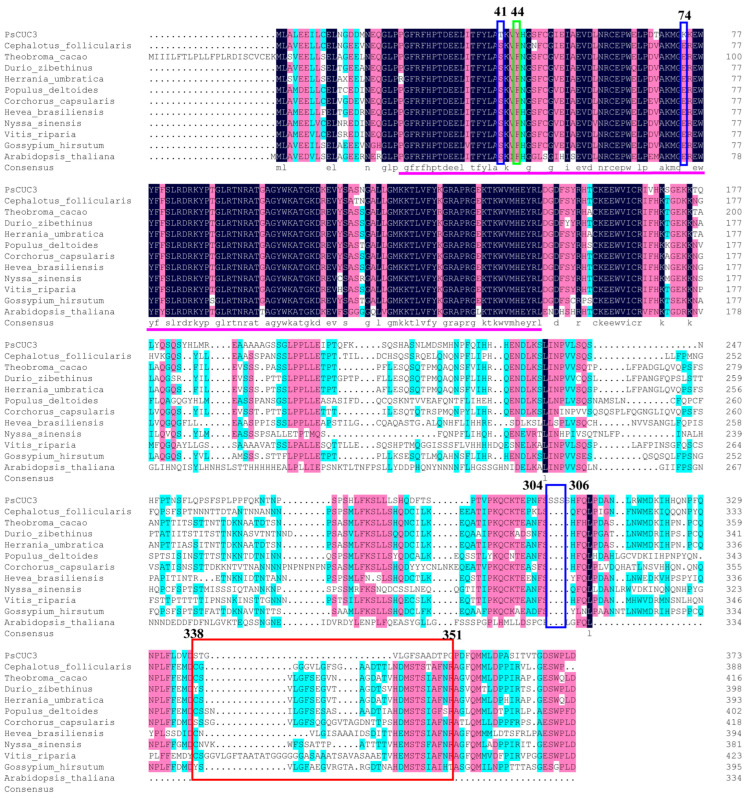
Alignment of the amino acid sequences of PsCUC3 with NAM/CUC3 proteins from 11 different plant species, including *Arabidopsis thaliana*. The NAC domain is highlighted in rose red. Boxes denote positions where conserved amino acids in PsCUC3 from ‘Doulv’ peony differ from those in other species.

Multiple sequence alignments of NAM/CUC3 proteins from various species—including *Paeonia suffruticosa* ‘Doulv’ (OP745631), *Cephalotus follicularis* (GAV65838.1), *Theobroma cacao* (XP_017981595.1), *Durio zibethinus* (XP_022769445.1), *Herrania umbratica* (XP_021284753.1), *Populus deltoides* (KAH8514964.1), *Corchorus capsularis* (OMO64504.1), *Hevea brasiliensis* (XP_021665943.1), *Nyssa sinensis* (KAA8537376.1), *Vitis riparia* (XP_034675316.1), *Gossypium hirsutum* (NP_001313889.1), and *Arabidopsis thaliana* (NM_106292.3)—revealed high sequence identity, particularly in the N-terminal regions, which were highly conserved across all examined NAM/CUC3 proteins (Figure 6).

The N-terminal region of the PsCUC3 protein, especially the NAC domain, demonstrated significant conservation. However, the C-terminal region of PsCUC3 exhibited high variability. Notably, some conserved amino acids in NAM/CUC3 proteins from other species underwent substitutions in PsCUC3 from ‘Doulv’ peony. Specifically, amino acids at positions 41, 44, and 74 changed from serine (S), phenylalanine (F), and glutamate (E) to threonine (T), tyrosine (Y), and lysine (K), respectively. Additionally, three serine residues (S) were inserted between positions 304 and 306 in PsCUC3. The amino acid sequence from positions 338 to 351 in the C-terminal region of PsCUC3 from ‘Doulv’ also showed clear differences compared to other species (Figure 6).

**Figure 7:**
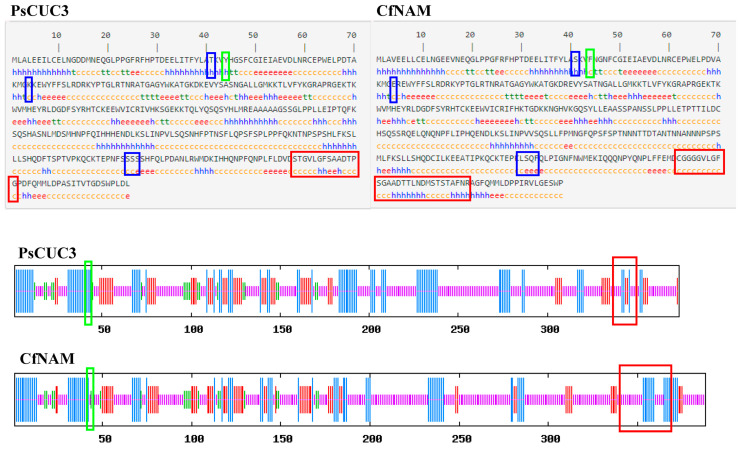
Protein secondary structures of PsCUC3 from ‘Doulv’ peony and CfNAM from *Cephalotus follicularis* were predicted using SOPMA. Blue boxes highlight regions where the secondary structures of PsCUC3 and CfNAM are identical. Green and red boxes denote regions where the secondary structures of PsCUC3 have altered. h: alpha helix; e: extended strand; t: beta turn; c: random coil.

Multiple sequence alignments revealed that PsCUC3 possesses the highest amino acid sequence similarity (65.83%) to the NAM domain-containing protein from *Cephalotus follicularis* (GAV65838.1). SOPMA was employed to predict the protein’s secondary structure. According to the analysis, alterations in conserved amino acids at positions 41, 74, and 304-306 in PsCUC3 did not affect its secondary structure. However, the modification at residue 44, where phenylalanine (F) in CfNAM of *Cephalotus follicularis* was replaced by tyrosine (Y) in PsCUC3, transitioned the structure from a random coil (c) to an alpha helix (h). Additionally, the amino acid sequence from residues 338-351 in the C-terminal region of PsCUC3 differed markedly from those of other NAM/CUC proteins, resulting in a significant reduction in alpha helix content (Figure 7).

### 2.5. Protein–Protein Interaction Analysis of PsCUC3

**Figure 8:**
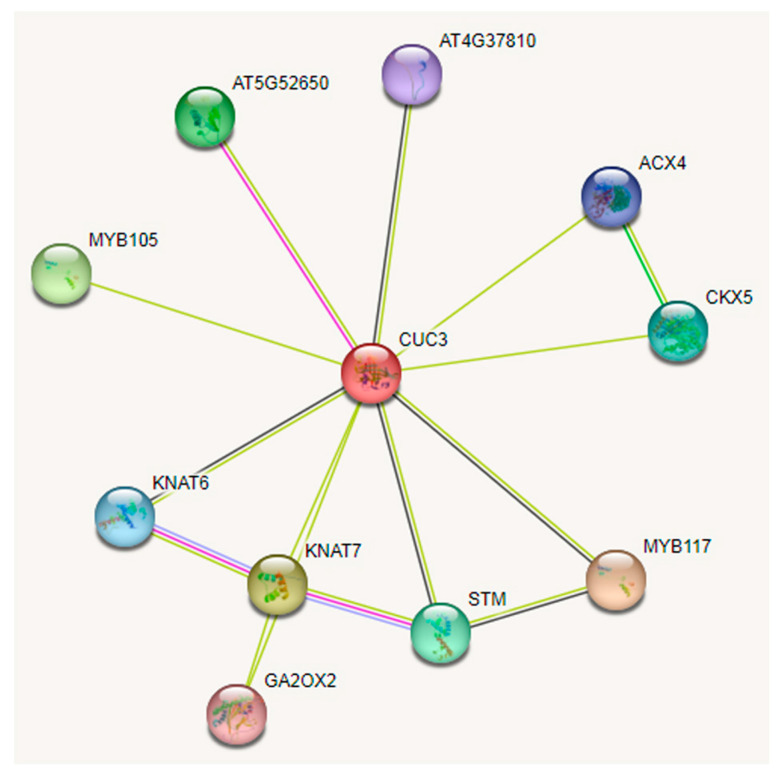
Interaction network analysis of CUC3 protein in *Arabidopsis thaliana*.

The phylogenetic analysis of PsCUC3 and Arabidopsis NAC proteins revealed that PsCUC3 is homologous to AtNAC31 (CUC3, AT1G76420) (Figure S2). STRING_v12.0 was utilized to predict the protein–protein interactions of CUC3 using the Arabidopsis protein database. As depicted in Figure 8, abCUC3 potentially interacts with ten proteins categorized into three functional groups: The first group is proteins associated with boundary specification, meristem initiation and maintenance, and organ patterning, including MYB105, MYB117, STM, and KNAT6. Notably, MYB117 also plays a role in floral organ development and ovule outgrowth initiation. The second group is proteins involved in structural formation and metabolism, such as AT5G52650 (structural constituent of ribosome), AT4G37810 (regulates epidermal patterning and stomatal patterning), ACX4 (acyl-coenzyme A oxidase 4, crucial for the initial step of peroxisomal fatty acid beta-oxidation in post-germinative growth of oilseed species), and KNAT7 (implicated in secondary cell wall biosynthesis and xylem development). The third group is proteins related to hormone regulation, including CKX5 (cytokinin oxidase/dehydrogenase, catalyzing cytokinin degradation) and GA2OX2 (Gibberellin 2-beta-dioxygenase 2, regulating endogenous gibberellin levels).

### 2.6. Chlorophyll Contents in the Petaloid Stamens of ‘Doulv’ and Expression Patterns of PsCUC3 Gene

**Figure 9:**
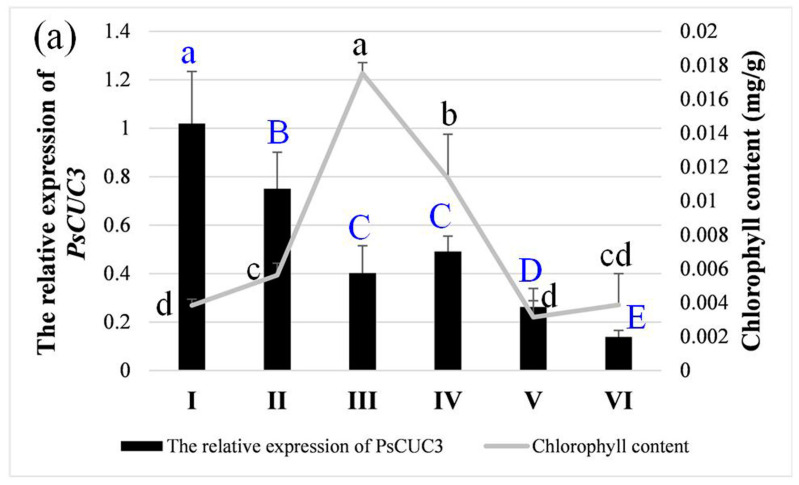

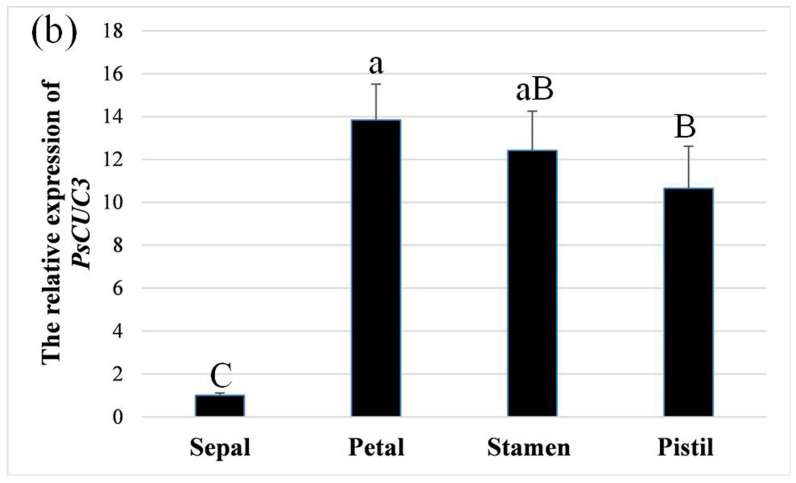
Chlorophyll contents and expression analysis of *PsCUC3*. (**a**) Chlorophyll contents and relative expression levels of *PsCUC3* in petaloid stamens at different developmental stages of ‘Doulv’. (**b**) Relative expression levels of *PsCUC3* in different tissues of ‘Doulv’ flower. Different letters indicate significant differences at *p* < 0.05 (lower case letters) or *p* < 0.01 (capital letters), respectively (based on the one-way ANOVA test).

The blooms of ‘Doulv’ peony exhibited green hues at Stage III, transitioning to white by Stage V (Figure 1). The primary floral components, the petaloid stamens, showed an increase in chlorophyll content, peaking at Stage III before significantly diminishing by Stages V and VI (Figure 9a). This chlorophyll profile aligns with the phenotypic changes observed in ‘Doulv’ peony. *GAPDH* and *ACT* were used as internal controls during various developmental stages in the petaloid stamens. Within these stamens, *PsCUC3* expression was highest at Stage I and declined progressively as the flowers opened (Figure 9a). These findings suggest no direct correlation between *PsCUC3* expression levels and changes in chlorophyll content in the petaloid stamens of ‘Doulv’. In different floral tissues, *UBC* and *MBF1A* acted as internal controls. The expression levels of *PsCUC3* were relatively uniform across the petals, petaloid stamens, and petaloid pistils, all displaying high expression levels, whereas its expression was markedly reduced in the sepals (Figure 9b).

### 2.7. Subcellular Localization of PsCUC3 Protein

**Figure 10:**
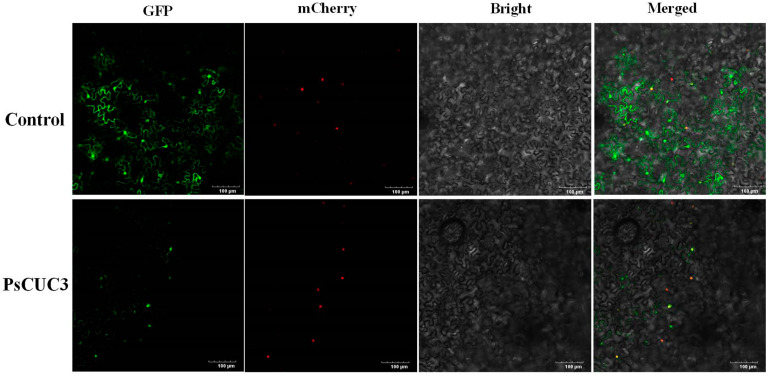
Subcellular localization analysis of PsCUC3 protein.

The PsCUC3 protein was anticipated to localize within the nucleus and potentially function in transcriptional activation and DNA binding. To validate these predictions, the subcellular localization of PsCUC3 was examined in tobacco (*Nicotiana benthamiana*) leaves through transient transformation using a super-promoter-driven GFP-PsCUC3 fusion protein. Additionally, mCherry was employed as a nuclear localization marker. Laser confocal microscopy (LSCM) revealed that GFP fluorescence signals from GFP-PsCUC3 were specifically localized in the nucleus. Similarly, the nuclear localization of mCherry, which shifted from red to yellow when merged with GFP signals, confirmed the nuclear presence. In contrast, control experiments using the empty super1300-GFP vector showed GFP localization in both the nucleus and the cytomembrane (Figure 10). These observations are consistent with predictions made by Plant-mPLoc, supporting the role of PsCUC3 as a transcription factor involved in regulating floral development in ‘Doulv’ peony.

## 3. Discussion

Tree peonies exhibit a broad array of varieties, each resulting from extensive hybridization during cultivar development, leading to a complex and somewhat obscure genetic background. Notably, chromosome-level genome assembly for *Paeonia ostii* and a draft genome for the *Paeonia suffruticosa* cultivar “Luo shen xiao chun” have been reported [12,24], highlighting the substantial genetic diversity across different cultivars of *Paeonia suffruticosa*. Prior studies focusing on reference genes in tree peonies have demonstrated the lack of universality among existing reference genes for qRT-PCR analysis across different peony varieties [13,14,15,16,17]. These findings underscore the absence of a universal reference gene suitable for all peony varieties, emphasizing the need to screen for appropriate reference genes when conducting gene expression analysis in previously unreported peony varieties.

Green flower peonies are considered precious and rare and are very popular in China. There are more than ten cultivars of green flower peonies in China, but none of them can stay green during flower opening; all their blooms transition to white or pink upon opening, diminishing their ornamental appeal. The molecular mechanism underlying flower chlorosis remains unclear.

‘Doulv’ peony is the most famous and representative cultivar of green flower peony in China. In this context, our study analyzed nine candidate reference genes in ‘Doulv’ peony. The results identified *GAPDH* and *ACT* as the most stable reference genes in the petaloid stamens across various developmental stages of ‘Doulv’, while *UBC* and *MBF1A* proved to be the most stable in different tissues of the ‘Doulv’ flower. The selection of appropriate reference genes was a necessary experimental foundation for the further study of the molecular mechanisms underlying flower chlorosis and flower type formation in ‘Doulv’ peony, providing technical support for prolonging the coloring time of ‘Doulv’ in practice, as well as improving its ornamental quality, which has high economic value and practical significance. Based on this experimental foundation, gene functional analysis could be performed for ‘Doulv’ peony.

NAC transcription factors constitute a plant-specific family initially identified in Petunia [18]. *Arabidopsis thaliana* harbors 105 genes within this family [25,26], while *Oryza sativa* contains 140 NAC or NAC-like genes [27]. NAC proteins are distinguished by their highly conserved N-terminal domains, comprising approximately 150 amino acids capable of binding both DNA and other proteins [28]. The highly variable C-terminal regions of these proteins play a critical role in transcriptional regulation [29,30]. NAC proteins are integral to a multitude of plant biological processes, including responses to abiotic stress [31,32], lignin biosynthesis [33,34], fruit ripening regulation [35,36], leaf senescence [37,38,39], pattern formation [18], organ separation [19], organ boundary formation [20], and flower development [21,22]. *CUC* genes, members of the NAC family, are named due to the cup-like appearance of the fused cotyledons they produce [18,19]. *Arabidopsis thaliana* expresses three such genes, which are implicated in embryonic, boundary, and shoot meristem formation, alongside leaf and flower development [18,19,20,21,22,40,41]. In Arabidopsis, while most single *cuc* mutants appear phenotypically normal, a minority exhibit heart-shaped cotyledons; however, double and triple mutants predominantly display cup-shaped cotyledons [19,20]. *CUC1*, *CUC2*, and *CUC3* demonstrate partial functional redundancy [40]. The expression of *CUC2* and *CUC3*, mediated by Arabidopsis histidine kinase 2 (AHK2) and AHK3, may regulate flower development through cytokinin enhancement [21]. Overexpression of *CUC3* in Arabidopsis results in compact flower arrangements and stunted inflorescences [21]. In Petunia, diminished expression of the *CUC3* ortholog *NH16* through virus-induced gene silencing (VIGS) leads to increased fusion between petals and stamens, as well as stamens and carpels, frequently resulting in the formation of additional petals [22].

The *PsCUC3* gene was selected for investigation based on previous studies suggesting its involvement in the floral development of ‘Doulv’ peony. This study involved gene cloning and expression analysis of *PsCUC3*. The results from qRT-PCR demonstrated no correlation between the expression levels of *PsCUC3* and changes in chlorophyll content within the petaloid stamens of ‘Doulv’. The expression levels of *PsCUC3* across petaloid tissues (petals, petaloid stamens, and petaloid pistils) displayed no significant variation, whereas a marked reduction was observed in the sepals. These findings suggest that the *PsCUC3* gene may play a role in petaloid tissue formation in ‘Doulv’ peony.

Multiple sequence alignments revealed that PsCUC3 is a homologous protein to the NAM/CUC3 proteins from other species. In Arabidopsis and Petunia, CUC3 is involved in boundary formation [18,19] and flower development [21,22]. Subcellular localization analysis showed that the PsCUC3 protein is localized in the nucleus. A mutation at amino acid position 44 in PsCUC3 led to alterations in the protein’s secondary structure; this residue is located within the NAC domain, which is capable of binding DNA and regulating gene expression. Changes at residues 338-351 in the C-terminal region of PsCUC3 resulted in a significant reduction in alpha helices, a feature of the transcriptional activation domain that may be involved in protein–protein interactions and transcriptional activation. Protein–protein interaction analysis suggested that CUC3 could interact with MYB105, MYB117, STM, and KNAT6, influencing boundary formation and organ patterning. Additionally, interactions with CKX2 and GA2OX2 indicate that CUC3 may also regulate growth and development by modulating hormone levels. PsCUC3 might act as a transcription factor, interacting with other proteins to regulate flower development in ‘Doulv’ peony.

## 4. Materials and Methods

### 4.1. Plant Materials

Five-year-old ‘Doulv’ peonies were cultivated at Shenzhou Peony Garden. Plants displaying uniform developmental progress were selected and labeled prior to blooming. The developmental stages of ‘Doulv’ peony flowers were categorized into six distinct phases based on the methodology described by Wang et al. [42]. Stage I involved the cracking of sepals, revealing the outer petals. In Stage II, the inner petals became visible, and the buds appeared fluffy. By Stage III, the outer petals had fully expanded, and the inner petals loosened, though the innermost petals remained closed. Stage IV saw the full expansion of the inner petals and the exposure of the carpels. During Stage V, the inner petals were completely expanded, with the carpels fully exposed. Finally, in Stage VI, the outer petals started to dehydrate and undergo senescence. Tissue samples, including petaloid stamens from Stages I through VI, as well as sepals, petals, petaloid stamens, and petaloid pistils from Stage III, were collected for further analysis.

### 4.2. RNA Extraction and Reverse Transcription

RNA extraction from ‘Doulv’ peony was performed using the MiniBEST Plant RNA Extraction Kit (TaKaRa Bio, Beijing, China). The integrity of the extracted RNA was verified via 1% (*w*/*v*) agarose gel electrophoresis, ensuring only samples with clear and distinct 28S/18S rRNA bands were selected for further analysis. Subsequent quantification was conducted using a Multiskan Go microplate spectrophotometer (Thermo Scientific, Waltham, MA, USA), with A_260_/A_280_ absorbance ratios ranging from 1.8 to 2.0, indicative of high-quality RNA. For reverse transcription, 1000 ng of RNA from each sample was utilized as a template in conjunction with a PrimeScript™ RT reagent Kit with gDNA Eraser (TaKaRa Bio, Beijing, China). The resulting cDNA samples were then stored at −20 °C for future use.

### 4.3. Selection of Candidate Reference Genes and Primer Design

Nine candidate reference genes were selected based on RNA-seq data from ‘Doulv’ and previous studies in other varieties of tree peony [13,14,15,16,17]. The genes selected for this study include *Actin* (*ACT*), *β-Tubulin* (*TUB*), *ubiquitin conjugating enzyme* (*UBC*), *ubiquitin* (*UBQ*), *ubiquitin protein ligase* (*UPL*), *protein phosphatase 2A* (*PP2A*), *protein phosphatase 2C* (*PP2C*), *multiprotein bridging factor 1A* (*MBF1A*) and *glyceraldehyde-3-phosphate dehydrogenase* (*GAPDH*). Primer design was carried out using Primer Premier 5.0. The primer sequences for *PP2C* were adopted from the work of Liu et al. [16]. Details of all primer sequences are provided in Table 1.

### 4.4. PCR Amplification and Amplification Efficiency for QRT-PCR

A mixed cDNA sample consisting of equal volumes from sepals, petals, petaloid stamens, and petaloid pistils at Stage III was utilized for PCR amplification. The reaction was set up in a final volume of 20 μL, containing 10 μL of 2 × Hieff^®^PCR Master Mix (With Dye) (Yeasen, Shanghai, China), 7.0 μL of ddH_2_O, 1.0 μL of the diluted mixed cDNA, 1.0 μL of forward primer (10 μM), and 1.0 μL of reverse primer (10 μM). The PCR cycling conditions were as follows: an initial denaturation at 94 °C for 5 min, followed by 35 cycles of 94 °C for 30 s, 60 °C for 30 s, and 72 °C for 20 s, with a final elongation step at 72 °C for 10 min. The PCR products were then visualized through 1.5% agarose gel electrophoresis.

For qRT-PCR analysis, the mixed cDNA was diluted in a five-fold gradient, and a standard curve was prepared with dilution factors of 1, 5, 5^2^, 5^3^, 5^4^, 5^5^. The qRT-PCR was performed on a LightCycler^®^ 96 system (Roche, Basel, BC, Switzerland). Each 10 μL reaction volume included 5.0 μL of TB Green™ Premix Ex Taq™ II (Tli RNaseH Plus) (TaKaRa), 3.0 μL of ddH_2_O, 1.0 μL of diluted mixed cDNA, 0.5 μL of forward primer (10 μM), and 0.5 μL of reverse primer (10 μM). The reaction conditions were set to 95 °C for 60 s, followed by 40 cycles of 95 °C for 10 s and 60 °C for 30 s. A melting curve analysis was conducted at the end of the cycles by heating from 95 °C for 10 s and 65 °C for 60 s to 97 °C at a gradual rate. Each sample was run in triplicate for technical accuracy, and ddH_2_O was included as a negative control. The amplification efficiency (E) and correlation coefficient (*R*^2^) were calculated based on the standard curve slope.

### 4.5. QRT-PCR Analysis

For qRT-PCR analysis, cDNAs from petaloid stamens of ‘Doulv’ peony at developmental Stages I through VI, along with sepals, petals, petaloid stamens, and petaloid pistils at Stage III, were utilized. The analysis was performed using a LightCycler^®^ 96 system (Roche). The reaction mixture and PCR cycling parameters followed those previously described in Section 4.4. Each sample was tested in triplicate to ensure technical reliability, and ddH_2_O was included as a negative control in parallel. Expression stability (M value, GeNorm_3.5), stability value (S value, NormFinder_v20), and standard deviation (SD, BestKeeper_v1.0) were used to evaluate the stability of the candidate genes. BestKeeper_v1.0 was used to calculate the coefficient of correlation (r), standard deviation (SD), and coefficient of variation (CV). If the SD value was greater than 1, the reference gene was unstable. If the SD value was less than 1, the stable reference gene had a larger r value, as well as smaller SD and CV values. A geomean of ranking values, derived from M values, S values, and SD, was utilized to comprehensively assess the expression stability of these genes. Additionally, paired difference analysis (V value) conducted by GeNorm_v3.5 determined the optimal number of internal control genes. The default V value is 0.15. If the V*n*/*n* + 1 value is less than 0.15, the optimal number of reference genes is n.

### 4.6. Gene Cloning and Bioinformatics Analysis of PsCUC3 in ‘Doulv’ Peony

The *PsCUC3* gene was selected for investigation based on its potential involvement in the floral development of ‘Doulv’ peony. Primer design was conducted using Primer Premier 5.0, with the sequences being 5′ TTCTCTCTCTGTCCATCTCTG 3′ for the forward primer and 5′ GCACTTTTCCACATTTACAGA 3′ for the reverse primer. The PCR reaction was carried out in a 50 μL mixture containing 25 μL of 2 × Phanta^®^ Flash Master Mix (Dye Plus) (Vazyme, Nanjing, China), 19.0 μL of ddH_2_O, 2.0 μL of diluted mixed cDNA, 2.0 μL of forward primer (10 μM), and 2.0 μL of reverse primer (10 μM). The PCR cycling conditions were set as follows: an initial denaturation at 98 °C for 30 s, followed by 35 cycles of 98 °C for 10 s, 52 °C for 5 s, and 72 °C for 15 s, with a final elongation step at 72 °C for 1 min. The resulting PCR product was resolved by 1.5% gel electrophoresis and subsequently extracted using a Gel Extraction Kit (CWBIO, Taizhou, China). For cloning, the *TaKaRa Ex Taq^®^* (TaKaRa) and dNTPs (TaKaRa) were used to add an adenine to the 3′ end of the *PsCUC3* PCR product. The A-tailed product was then cloned into the pMD™ 19-T Vector (TaKaRa), and the ligation product was transformed into *E. coli* DH5α competent cells (TaKaRa). Positive clones were identified through PCR screening and subsequently sent for sequencing at Shanghai Sangon Biological Engineering Technology & Services, Shanghai, China.

An ORF search was performed according to the NCBI ORF Finder (https://www.ncbi.nlm.nih.gov/orffinder/, accessed on 6 October 2022), and conserved domain analysis was carried out using NCBI Conserved Domains Search (https://www.ncbi.nlm.nih.gov/Structure/cdd/wrpsb.cgi?, accessed on 6 October 2022). The properties of PsCUC3 were analyzed on ExPASy (https://web.expasy.org/protparam/, accessed on 6 October 2022). The protein secondary structure of PsCUC3 was predicted by SOPMA (https://npsa-prabi.ibcp.fr/cgi-bin/npsa_automat.pl?page=npsa_sopma.html, accessed on 6 October 2022). Subcellular localization of PsCUC3 was analyzed on Plant-mPLoc (http://www.csbio.sjtu.edu.cn/bioinf/plant-multi/, accessed on 6 October 2022). A homology search was performed according to NCBI-BLAST (https://blast.ncbi.nlm.nih.gov/Blast.cgi, accessed on 6 October 2022), and DNAMAN6.0 software was used for homology comparison. The annotated Arabidopsis NAC proteins were downloaded from PlantTFDB (https://planttfdb.gao-lab.org/, accessed on 6 October 2022), and the phylogenetic tree of PsCUC3 and Arabidopsis NAC proteins was inferred using MEGA-X with the neighbor-joining method. Bootstrap values were calculated for 1000 iterations. The protein–protein interaction network was analyzed by STRING_v12.0 (https://cn.string-db.org/, accessed on 6 October 2022).

### 4.7. Chlorophyll Content Determination of Petaloid Stamens in ‘Doulv’ Peony

The petaloid stamens of ‘Doulv’ peony at developmental Stages I through VI were harvested for chlorophyll content analysis. Fresh samples were pulverized in liquid nitrogen and subsequently extracted with 5 mL of 95% ethanol for 48 h under dark conditions. Following the extraction, samples were centrifuged at 10,000 rpm for 10 min, and the absorbance of the supernatants was measured at 665 nm and 649 nm wavelengths. Chlorophyll (Chl) concentrations were calculated using the following equation: C_Chl_ (mg/L) = 18.16A_649_ + 6.63A_665_. The total chlorophyll content was then calculated using the following formula: Chl content (mg/g) = C_Chl_ (mg/L) × V (L)/FW (g), where C_Chl_ represents the chlorophyll concentration, V is the volume of the extract, and FW is the fresh weight of the samples. Quantitative analysis was performed on three individual plants. Data analysis, including statistical evaluations, was conducted using Microsoft Excel. Differences in chlorophyll content across the different stages were assessed using a one-way ANOVA test.

### 4.8. Gene Expression Analysis

The expression pattern of the *PsCUC3* gene was investigated using qRT-PCR, conducted on a LightCycler 96 system (Roche, Germany). Primer sequences for qRT-PCR were designed using Primer Premier 5.0, with the forward primer being 5′ TGCTGGTTCGTCTGGCTTG 3′ and the reverse primer 5′ AGGTGAAAACGAGGGCTGGAG 3′. *GAPDH* and *ACT* served as internal controls for assessing expression in the petaloid stamens at different developmental stages of the ‘Doulv’ flowers. For other tissues of the ‘Doulv’ flower, *UBC* and *MBF1A* were utilized as internal controls. The reaction mixture composition and PCR cycle parameters followed those previously described in Section 4.4. Relative expression levels of *PsCUC3* were calculated using the 2^−ΔΔCt^ method as described by Schmittgen and Livak [43]. The expression levels in petaloid stamens at Stage I and in sepals at Stage III were used as reference controls. Analysis was performed on three individual plants, with each reaction run in triplicate. Statistical analyses were conducted using Microsoft Excel, employing a one-way ANOVA test to evaluate the data.

### 4.9. Subcellular Localization Analysis of the PsCUC3 Protein

The full-length coding region of *PsCUC3* was successfully cloned using specific primers designed to include restriction enzyme sites: the forward primer was CGGGGTACCCCGTTCTCTCTCTGTCCATCTCTG (*Kpn* I site underlined), and the reverse primer was CCGGAATTCCGGGCACTTTTCCACATTTACAGA (*EcoR* I site underlined). This *PsCUC3* fragment was subsequently inserted into the super1300-GFP vector using a restriction-enzyme-mediated cloning method. This construct, driven by a super-promoter, resulted in the super-GFP-PsCUC3 construct. For control purposes, the empty super1300-GFP vector was employed, and p2300-35S-*H2B*-*mcherry* was utilized to confirm nuclear localization. These plasmids were transformed into *Agrobacterium tumefaciens* GV3101 (Biomed). For transient expression, an *Agrobacterium* strain containing the super-GFP-PsCUC3 construct and another containing p2300-35S-*H2B-mcherry*, both at an optical density (OD_600_) of 0.3, were mixed in equal volumes. A similar procedure was followed for the strain containing the empty super1300-GFP vector. The *Agrobacterium* mixtures were incubated under dark conditions for 3 h and then injected into tobacco (*Nicotiana benthamiana*) leaves. Post-infiltration, the transformed tobacco plants were kept under dark conditions for 48–72 h. Subsequently, the leaves were examined using an FV3000 laser confocal microscope (LSCM, Olympus, Tokyo, Japan) to visualize and capture the GFP and mCherry fluorescence signals, providing insights into the subcellular localization and expression dynamics of the PsCUC3 protein within plant cells.

## 5. Conclusions

In this study, nine candidate reference genes were selected based on published literature and transcriptome sequencing data. Their expression stability was comprehensively evaluated using GeNorm_v3.5, NormFinder_v20, and BestKeeper_v1.0 software. *GAPDH* and *ACT* were identified as the most stable reference genes in the petaloid stamens across various developmental stages of ‘Doulv’, while *UBC* and *MBF1A* were determined to be the optimal reference genes in different tissues of ‘Doulv’ flowers. A robust qRT-PCR detection system for ‘Doulv’ peony was successfully established, enhancing future gene function studies in this cultivar. Gene cloning of *PsCUC3* was conducted, revealing that some conserved amino acids in the PsCUC3 protein from ‘Doulv’ peony differed from those in NAM/CUC3 proteins from other species, resulting in alterations to its protein secondary structure. QRT-PCR results indicated that *PsCUC3* expression did not correlate with changes in chlorophyll content in the petaloid stamens at different developmental stages of ‘Doulv’. However, its expression was associated with the formation of petaloid organs in ‘Doulv’ flowers. Subcellular localization analysis demonstrated that the PsCUC3 protein was localized in the nucleus, suggesting its potential role as a transcription factor.

In summary, the findings suggest that the *PsCUC3* gene may be involved in the floral type mutation of ‘Doulv’ peony. Further research is needed to elucidate the molecular and physiological mechanisms underlying this association.

## Figures and Tables

**Table 1:** Primers used for qRT-PCR.

Reference Gene	Primer Sequence (5′-3′)	Product Length	Tm/°C	Efficiency	*R* ^2^
*ACT*	F: GAGAGATTCCGTTGCCCTGA	211	60	99.88%	0.9979
	R: TAGGTGCAAGAGCCGTGATT				
*TUB*	F: CATTTCTTCATGGTTGGGTTCG	183	62	101.57%	0.9928
	R: CTTTGTGCTCATCTTTCCTCGG				
*UPL*	F: CTTTATCACCTCCATTGTTTCGC	207	60	No band	
	R: ACACTATTTGTTTCCACCCGTCT				
*PP2A*	F: TGTTTGGATGTTCTCAAGGCAG	147	60	165.38%	0.9911
	R: CAGAATTATCCGCTCCTCGTC				
*PP2C*	F: GTTGGTGGCGTTCTTGCTGTTT	233	63	96.76%	0.9949
	R: CCTCGTGCGTAGGCTTCTTGTAT				
*MBF1A*	F: GCGTTGACGACTTTCTCATCCTTC	140	63	97.04%	0.9976
	R: CGCACATCCTTGACAAACCCTA				
*GAPDH*	F: TGTTCACTCCATCACTGCTAC	189	55	98.70%	0.9968
	R: ACATCCACAGTAGGAACACGA				
*UBQ*	F: AAGACGCTGACTGGCAAGACA	199	61	102.94%	0.9973
	R: GCAAGACGAGATGAAGGGTAGACT				
*UBC*	F: ACCTCCCGAAACTCTCTATGACG	171	56	90.63%	0.9962
	R: GGGTCTTCACCAGGAGGATGTAG				

**Table 2:** The comprehensive analysis of stability of candidate reference genes in petaloid stamens at different developmental stages of ‘Doulv’.

Reference Gene	GeNorm M Value	NormFinder S Value	BestKeeper SD	Geomean of Ranking Values	Ranking
*GAPDH*	0.567	0.132	0.96	0.416	1
*ACT*	0.661	0.205	0.98	0.510	2
*UBC*	0.638	0.271	0.91	0.540	3
*UBQ*	0.682	0.308	0.98	0.590	4
*MBF1A*	0.824	0.487	0.79	0.682	5
*PP2C*	0.734	0.323	1.39	0.691	SD > 1
*TUB*	1.102	0.723	1.55	1.073	SD > 1

**Table 3:** The comprehensive analysis of stability of candidate reference genes in different tissues of ‘Doulv’ flower.

Reference Gene	GeNorm M Value	NormFinder S Value	BestKeeper SD	Geomean of Ranking Values	Ranking
*UBC*	0.73	0.058	0.28	0.228	1
*MBF1A*	0.723	0.132	0.33	0.316	2
*PP2C*	0.788	0.356	0.24	0.407	r = 0.034
*UBQ*	0.931	0.533	0.14	0.411	r = −0.315
*ACT*	0.898	0.162	0.84	0.496	3
*GAPDH*	0.868	0.244	0.69	0.527	4
*TUB*	2.248	1.538	1.74	1.819	M value > 1.5, SD > 1

## Data Availability

Data are contained within the article and Supplementary Materials.

## Supplementary Materials

The following supporting information can be downloaded at https://www.mdpi.com/article/10.3390/plants13131741/s1: Figure S1: PCR product of *PsCUC3*; Figure S2: Phylogenetic analysis of PsCUC3 and Arabidopsis NAC proteins.
