# Supplementary material for: Selection of Stable Reference Genes for QRT-PCR in Tree Peony ‘Doulv’ and Functional Analysis of PsCUC3
Source: Plants (Basel). 2024 Jun 24;13(13):1741. doi: 10.3390/plants13131741 (PMC11243599; doi:10.3390/plants13131741)
Supplement: Supplementary file 1 [file plants-13-01741-s001.zip › plants-3035401-supplementary.pdf]

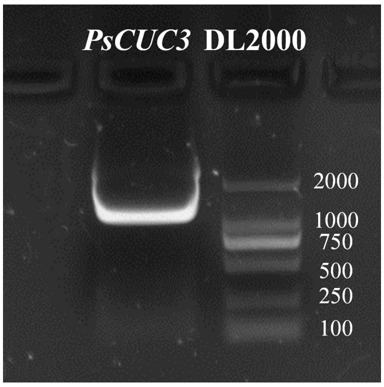

Figure S1. PCR product of *PsCUC3*.

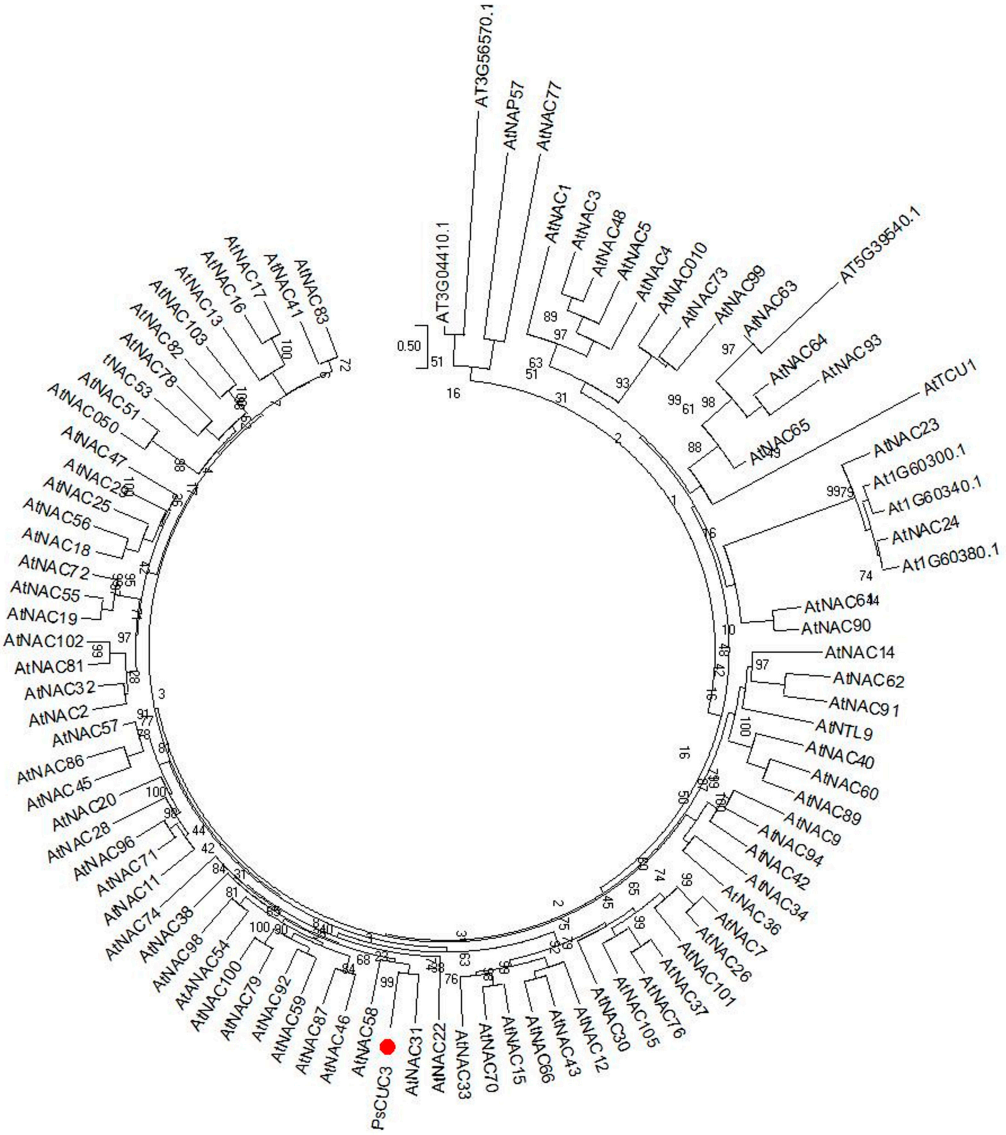

Figure S2. Phylogenetic analysis of PsCUC3 and Arabidopsis NAC proteins.
